# Supplementary material for: Misconceptions and Knowledge Gaps on Antibiotic Use and Resistance in Four Healthcare Settings and Five European Countries—A Modified Delphi Study
Source: Antibiotics (Basel). 2023 Sep 11;12(9):1435. doi: 10.3390/antibiotics12091435 (PMC10525245; doi:10.3390/antibiotics12091435)
Supplement: Supplementary file 1 [file antibiotics-12-01435-s001.zip › Supplementary file S1.pdf]

Supplementary file S1 – List of publications used for the extraction of the statements used in the Delphi study

1. Dempsey, P.P.; Businger, A.C.; Whaley, L.E.; Gagne, J.J.; Linder, J.A. Primary Care Clinicians' Perceptions about Antibiotic Prescribing for Acute Bronchitis: A Qualitative Study. *BMC Fam Pract* **2014**, *15*, doi:10.1186/S12875-014-0194-5.
2. Rusic, D.; Bukić, J.; Seselja Perisin, A.; Leskur, D.; Modun, D.; Petric, A.; Vilovic, M.; Bozic, J. Are We Making the Most of Community Pharmacies? Implementation of Antimicrobial Stewardship Measures in Community Pharmacies: A Narrative Review. *Antibiotics 2021, Vol. 10, Page 63* **2021**, *10*, 63, doi:10.3390/ANTIBIOTICS10010063.
3. van der Velden, A.; Duerden, M.G.; Bell, J.; Oxford, J.S.; Altiner, A.; Kozlov, R.; Sessa, A.; Pignatari, A.C.; Essack, S.Y. Prescriber and Patient Responsibilities in Treatment of Acute Respiratory Tract Infections — Essential for Conservation of Antibiotics. *Antibiotics* **2013**, *2*, 316, doi:10.3390/ANTIBIOTICS2020316.
4. Panagakou, S.G.; Spyridis, ikos; Papaevangelou, V.; Theodoridou, K.M.; Goutziana, G.P.; Theodoridou, M.N.; Syrogiannopoulos, G.A.; Hadjichristodoulou, C.S. Antibiotic Use for Upper Respiratory Tract Infections in Children: A Cross-Sectional Survey of Knowledge, Attitudes, and Practices (KAP) of Parents in Greece. *BMC Pediatr* **2011**, *11*, doi:10.1186/1471-2431-11-60.
5. Woods, C.J.; Morrice, Z.; Francis, N.A.; Little, P.; Verheij, T.; Leydon, G.M. Parent and Clinician Views of Managing Children with Symptoms of a Lower Respiratory Tract Infection and Their Influence upon Decisions to Take Part in a Placebo-Controlled Randomised Control Trial. *Antibiotics* **2021**, *10*, 356, doi:10.3390/ANTIBIOTICS10040356/S1.
6. Cordoba, G.; Siersma, V.; Lopez-Valcarcel, B.; Bjerrum, L.; Llor, C.; Aabenhus, R.; Makela, M. Prescribing Style and Variation in Antibiotic Prescriptions for Sore Throat: Cross-Sectional Study across Six Countries. *BMC Fam Pract* **2015**, *16*, 1–8, doi:10.1186/S12875-015-0224-Y/FIGURES/2.
7. Chaaban, T.; Ahouah, M.; Lombrail, P.; Le Febvre, H.; Mourad, A.; Morvillers, J.M.; Rothan-Tondeur, M. Decisional Issues in Antibiotic Prescribing in French Nursing Homes: An Ethnographic Study. *J Public Health Res* **2019**, *8*, 90–95, doi:10.4081/JPHR.2019.1533.
8. Willems, C.S.J.; Van den broek d'obrenan, J.; Numans, M.E.; Verheij, T.J.M.; Van der velden, A.W. Cystitis: Antibiotic Prescribing, Consultation, Attitudes and Opinions. *Fam Pract* **2014**, *31*, 149–155, doi:10.1093/FAMPRA/CMT077.
9. Björkman, I.; Berg, J.; Viberg, N.; Stålsby Lundborg, C. Awareness of Antibiotic Resistance and Antibiotic Prescribing in UTI Treatment: A Qualitative Study among Primary Care Physicians in Sweden. *Scand J Prim Health Care* **2013**, *31*, 50–55, doi:10.3109/02813432.2012.751695.
10. Plate, A.; Kronenberg, A.; Risch, M.; Mueller, Y.; Di Gangi, S.; Rosemann, T.; Senn, O. Treatment of Urinary Tract Infections in Swiss Primary Care: Quality and Determinants of Antibiotic Prescribing. *BMC Fam Pract* **2020**, *21*, 1–9, doi:10.1186/S12875-020-01201-1/TABLES/4.

11. Lakhani, J.D.; Lakhani, S.J.; Meera, S.; Sanket, P.; Sandeep, J. Appropriate Use of Antimicrobial Agents in Urinary Tract Infections: Perception of Physicians and Resident Doctors. *Journal of Integrated Health Sciences* **2019**, *7*, 19, doi:10.4103/JIHS.JIHS_18_19.

12. Duane, S.; Domegan, C.; Callan, A.; Galvin, S.; Cormican, M.; Bennett, K.; Murphy, A.W.; Vellinga, A. Using Qualitative Insights to Change Practice: Exploring the Culture of Antibiotic Prescribing and Consumption for Urinary Tract Infections. *BMJ Open* **2016**, *6*, e008894, doi:10.1136/BMJOPEN-2015-008894.

13. Leydon, G.M.; Turner, S.; Smith, H.; Little, P. Women's Views about Management and Cause of Urinary Tract Infection: Qualitative Interview Study. *BMJ* **2010**, *340*, 407, doi:10.1136/BMJ.C279.

14. Leydon, G.M.; Turner, S.; Smith, H.; Little, P. The Journey from Self-Care to GP Care: A Qualitative Interview Study of Women Presenting with Symptoms of Urinary Tract Infection. *The British Journal of General Practice* **2009**, *59*, e219, doi:10.3399/BJGP09X453459.

15. Minejima, E.; Lee, E.; Quach, S.; Santos, N.; Lou, M.; Wong-Beringer, A. Understanding Patient Perceptions and Attitudes toward Urinary Tract Infections and Treatment in a Medically Underserved Population. *Journal of the American College of Clinical Pharmacy* **2019**, *2*, 616–622, doi:10.1002/JAC5.1071.

16. Roope, L.S.J.; Tonkin-Crine, S.; Butler, C.C.; Crook, D.; Peto, T.; Peters, M.; Walker, A.S.; Wordsworth, S. Reducing Demand for Antibiotic Prescriptions: Evidence from an Online Survey of the General Public on the Interaction between Preferences, Beliefs and Information, United Kingdom, 2015. *Euro Surveill* **2018**, *23*, doi:10.2807/1560-7917.ES.2018.23.25.1700424.

17. Robert, A.; Nguyen, Y.; Bajolet, O.; Vuillemin, B.; Defoin, B.; Vernet-Garnier, V.; Drame, M.; Bani-Sadr, F. Knowledge of Antibiotics and Antibiotic Resistance in Patients Followed by Family Physicians. *Med Mal Infect* **2017**, *47*, 142–151, doi:10.1016/J.MEDMAL.2016.10.003.

18. McNulty, C.A.M.; Nichols, T.; Boyle, P.J.; Woodhead, M.; Davey, P. The English Antibiotic Awareness Campaigns: Did They Change the Public's Knowledge of and Attitudes to Antibiotic Use? *J Antimicrob Chemother* **2010**, *65*, 1526–1533, doi:10.1093/JAC/DKQ126.

19. Mcnulty, C.A.M.; Collin, S.M.; Cooper, E.; Lecky, D.M.; Butler, C.C. Public Understanding and Use of Antibiotics in England: Findings from a Household Survey in 2017. *BMJ Open* **2019**, *9*, doi:10.1136/BMJOPEN-2019-030845.

20. Van Hecke, O.; Butler, C.C.; Wang, K.; Tonkin-Crine, S. Parents' Perceptions of Antibiotic Use and Antibiotic Resistance (PAUSE): A Qualitative Interview Study. *J Antimicrob Chemother* **2019**, *74*, 1741–1747, doi:10.1093/JAC/DKZ091.

21. Hawke, K.L.; McGuire, T.M.; Ranmuthugala, G.; Van Driel, M.L. What Do Consumers Want to Know about Antibiotics? Analysis of a Medicines Call Centre Database. *Fam Pract* **2016**, *33*, 75–81, doi:10.1093/FAMPRA/CMV083.

22. Pavydė, E.; Veikutis, V.; Mačiulienė, A.; Mačiulis, V.; Petrikonis, K.; Stankevičius, E. Public Knowledge, Beliefs and Behavior on Antibiotic Use and Self-Medication in Lithuania. *Int J Environ Res Public Health* **2015**, *12*, 7002–7016, doi:10.3390/IJERPH120607002.

23. McCullough, A.R.; Parekh, S.; Rathbone, J.; Del Mar, C.B.; Hoffmann, T.C. A Systematic Review of the Public's Knowledge and Beliefs about Antibiotic Resistance. *J Antimicrob Chemother* **2016**, *71*, 27–33, doi:10.1093/JAC/DKV310.

24. Godycki-Cwirko, M.; Cals, J.W.L.; Francis, N.; Verheij, T.; Butler, C.C.; Goossens, H.; Zakowska, I.; Panasiuk, L. Public Beliefs on Antibiotics and Symptoms of Respiratory Tract Infections among Rural and Urban Population in Poland: A Questionnaire Study. *PLoS One* **2014**, *9*, e109248, doi:10.1371/JOURNAL.PONE.0109248.

25. Cals, J.W.L.; Boumans, D.; Lardinois, R.J.M.; Gonzales, R.; Hopstaken, R.M.; Butler, C.C.; Dinant, G.J. Public Beliefs on Antibiotics and Respiratory Tract Infections: An Internet-Based Questionnaire Study. *Br J Gen Pract* **2007**, *57*, 942–947, doi:10.3399/096016407782605027.

26. Roope, L.S.J.; Tonkin-Crine, S.; Herd, N.; Michie, S.; Pouwels, K.B.; Castro-Sanchez, E.; Sallis, A.; Hopkins, S.; Robotham, J. V.; Crook, D.W.; et al. Reducing Expectations for Antibiotics in Primary Care: A Randomised Experiment to Test the Response to Fear-Based Messages about Antimicrobial Resistance. *BMC Med* **2020**, *18*, doi:10.1186/S12916-020-01553-6.

27. Richmond, J.; Mangrum, R.; Wang, G.; Maurer, M.; Sofaer, S.; Yang, M.; Carman, K.L. An Informed Public's Views on Reducing Antibiotic Overuse. *Health Serv Res* **2019**, *54*, 1283, doi:10.1111/1475-6773.13175.
