# Supplementary material for: Misconceptions and Knowledge Gaps on Antibiotic Use and Resistance in Four Healthcare Settings and Five European Countries—A Modified Delphi Study
Source: Antibiotics (Basel). 2023 Sep 11;12(9):1435. doi: 10.3390/antibiotics12091435 (PMC10525245; doi:10.3390/antibiotics12091435)
Supplement: Supplementary file 1 [file antibiotics-12-01435-s001.zip › Supplementary file S2.pdf]

Supplementary file S2 - Educational tools

1. Antibiotic Free Prescription
2. Poster in Consultation Room or Leaflet: Viruses or Bacteria: What Made you Sick?
3. Urinary Tract Infections: A Leaflet for Older Adults and Their Families
4. Poster: 5 Myths about Urinary Tract Infections in Nursing Home Residents
5. Checklist for Pharmacists: What you Need to Know if You Have Been Prescribed an Antibiotic
6. Information cards about Antimicrobial Resistance and the Use of Antibiotics

HAPPY PATIENT

Antibiotic free prescription

Date: ____________

Patient name: ____

Infection	Most people get better by	Most common symptoms
☐ COVID-19	1 - 3 weeks	Fever, headache, loss of taste and/or smell
☐ Common cold	1 - 2 weeks	Runny nose, congestion, sore throat, dry cough
☐ Flu	1 - 2 weeks	Fever, shivering, muscle pain, cough
☐ Laryngitis	1 - 2 weeks	Dysphonia (hoarseness), sore throat, dry cough
☐ Pharyngitis / Tonsilitis	1 week	Fever, sore throat symptoms: pain while swallowing, enlarged and painful glands
☐ Acute bronchitis	2 - 4 weeks	Wheezing, cough with or without phlegm
☐ Sinusitis	1 - 2 weeks	Facial pain, fever, runny nose, congestion
☐ Acute middle ear infection	< 1 week	Ear pain, fever
☐ Exacerbation of COPD	1 - 2 weeks	Worsening of symptoms of COPD

You probably have a self-limiting infection and that is why you have not been prescribed an antibiotic. Antibiotics are not effective in treating viral infections. If given when not needed, antibiotics can be harmful as they lead to antimicrobial resistance. This means that antibiotics become ineffective and infections become increasingly difficult to treat.

When you have an infection, it is very important to get plenty of rest and give your body time to fight it off. If you follow these instructions, you should feel better soon:

- ☐ Get plenty of rest and consider staying at home to prevent the spread of infection
- ☐ Remember to drink a sufficient amount of fluids to avoid dehydration
- ☐ Wash your hands frequently
- ☐ You can take over-the-counter medication to alleviate your symptoms as recommended below by your healthcare provider

To alleviate symptoms

- ☐ For fever and aches ____
- ☐ For sore throat ____
- ☐ For nasal congestion ____
- ☐ Other: ____

Please, return to your healthcare provider if:

- ☐ Your symptoms get worse / do not improve in ______ day(s)
- ☐ You develop a high fever
- ☐ Other: ______________________________

The Prescriber

CONTACT: ______________________

Co-funded by the Health Programme of the European Union

This project is funded by the European Union's Third Health Programme (2014-2020). The content of this tool represents the views of the author only and is his/her sole responsibility; it cannot be considered to reflect the views of the European Commission and/or the Health and Digital Executive Agency (HaDEA), replacing the former CHAFEA since 01 April 2021, or any other body of the European Union. The European Commission and the Agency do not accept any responsibility for use that may be made of the information it contains.

HAPPY PATIENT

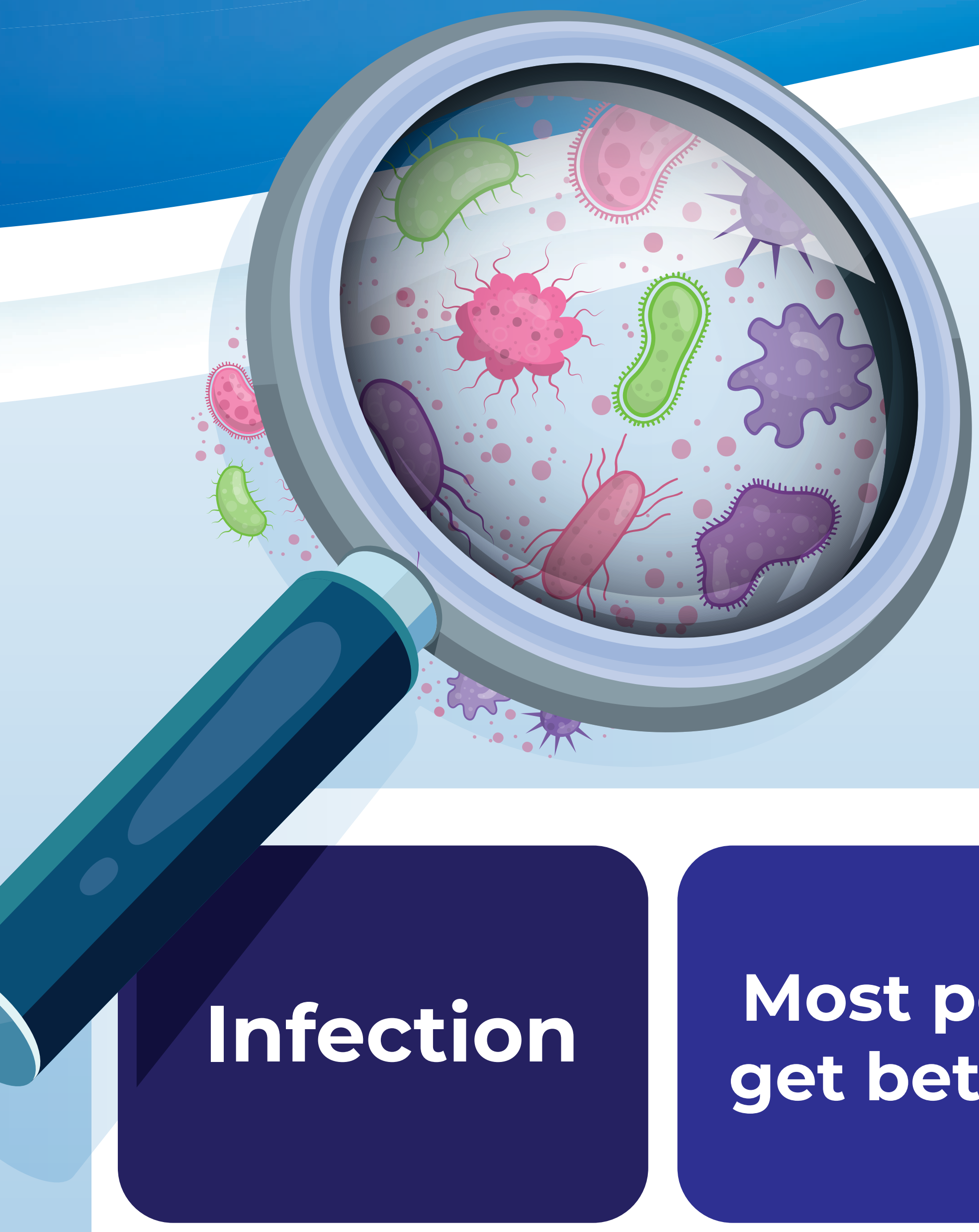

Viruses or bacteria

What caused your infection?

Infection	Most people get better by	Common cause			Are antibiotics recommended?
		Virus	Virus or bacteria	Bacteria	
COVID -19	1-3 weeks	✓			NO
Common cold	1-2 weeks	✓			NO
Flu	1-2 weeks	✓			NO
Laryngitis	1-2 weeks	✓			NO
Acute bronchitis / Bronchiolitis	2-4 weeks	✓			NO
Tonsilitis / Pharyngitis	1 week		✓		SOMETIMES
Sinusitis	1-2 weeks		✓		SOMETIMES
Acute middle ear infection	<1 week		✓		SOMETIMES
Exacerbation of COPD	1-2 weeks		✓		SOMETIMES
Pneumonia	1-2 weeks			✓	YES
Urinary tract infection	2-3 days			✓	YES

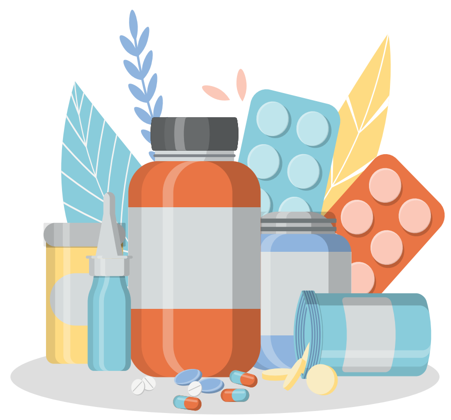

Antibiotics are only needed to treat certain infections caused by bacteria.
Viral infections should not be treated with antibiotics.
Use of antibiotics can lead to antimicrobial resistance.
This means that antibiotics become ineffective and infections become increasingly difficult to treat.

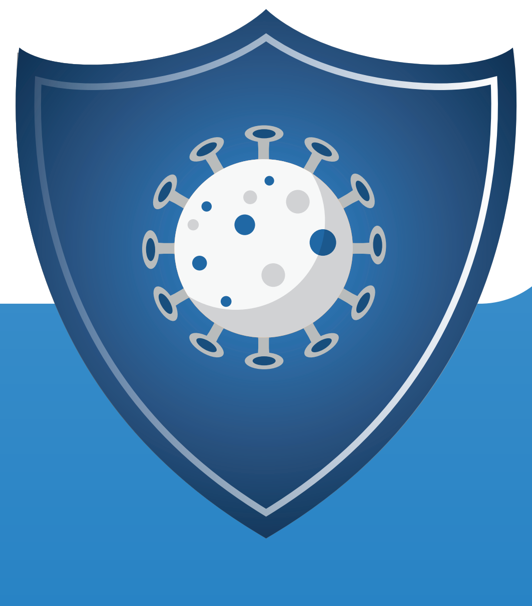

3rd Health Programme

Co-funded by the Health Programme of the European Union

This project is funded by the European Union's Third Health Programme (2014-2020).
The content of this poster represents the views of the author only and is his/her sole responsibility; it cannot be considered to reflect the views of the European Commission and/or the Health and Digital Executive Agency (HaDEA), replacing the former CHAFEA since 01 April 2021, or any other body of the European Union. The European Commission and the Agency do not accept any responsibility for use that may be made of the information it contains.

HAPPY PATIENT

URINARY TRACT INFECTIONS

A leaflet for older adults and their families

WHAT IS A URINARY TRACT INFECTION?

A urinary tract infection occurs when bacteria enter the urinary tract and cause symptoms. The bacteria involved in a urinary tract infection come from your own gastrointestinal tract, where they normally exist.

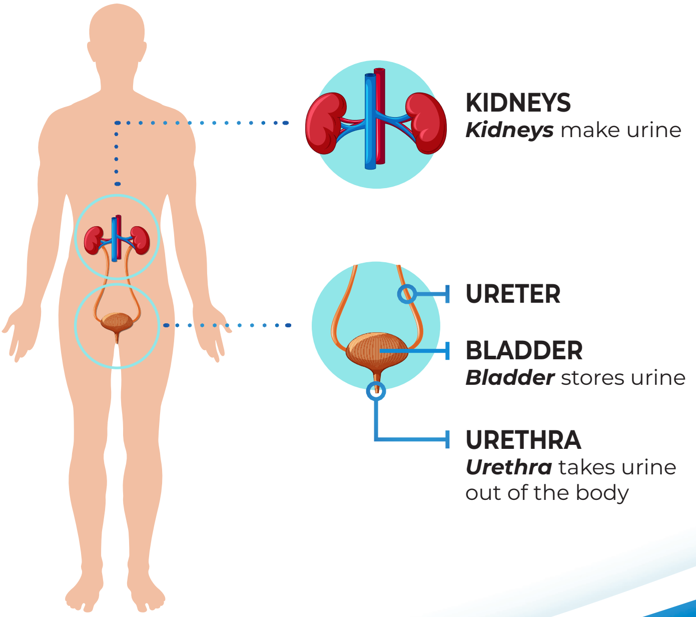

THE HEALTH ALLIANCE FOR PRUDENT PRESCRIPTION AND YIELD OF ANTIBIOTICS FROM A PATIENT-CENTRED PERSPECTIVE

This project was funded by the European Union's Health Programm (2014-2020)

Co-funded by the Health Programme of the European Union

WHAT ARE THE TYPICAL **SYMPTOMS** OF A URINARY TRACT INFECTION?

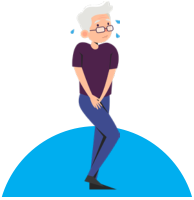

Frequent urination or urge

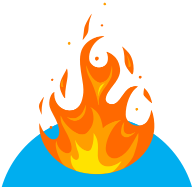

Burning sensation or pain when urinating

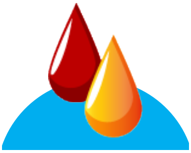

Blood in urine

- If you have some of these symptoms, you may have a urinary tract infection and you should contact a nurse or a medical doctor.
- If your symptoms do not improve within a couple of days after starting antibiotics, contact your doctor/nurse.

WHAT ARE THE **SYMPTOMS** OF A SERIOUS INFECTION?

If the symptoms above are followed by symptoms below, it may be due to a possibly serious infection or complication and you should contact a healthcare professional urgently:

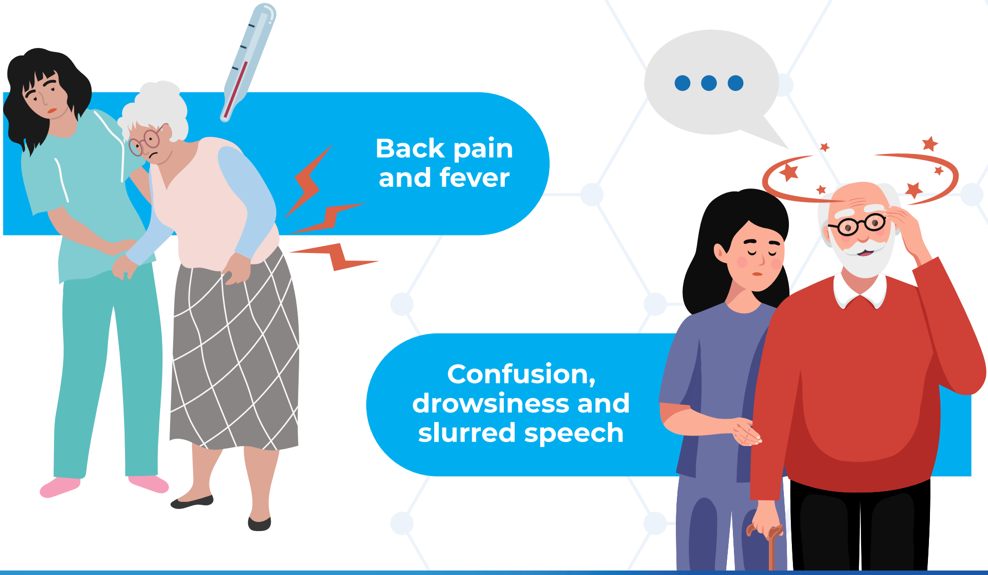

The content of this leaflet represents the views of the author only and is his/her sole responsibility; it cannot be considered to reflect the views of the European Commission and/or the Consumers, Health, Agriculture and Food Executive Agency (CHAFEA) or any other body of the European Union. The European Commission and the Agency do not accept any responsibility for use that may be made of the information it contains

ANTIBIOTICS FOR URINARY TRACT INFECTIONS: **PROS AND CONS**

Antibiotics shorten the duration of urinary symptoms and are life saving by preventing serious complications such as pyelonephritis and septicemia.

Antibiotics can come with side effects. Among the most common ones are rashes, vomiting, diarrhea, and increased risk of yeast infections.

Taking antibiotics increases the risk of resistant bacteria. Bacteria resistant to antibiotics do not respond well to treatment.

WHAT CAN YOU DO **TO PREVENT** A URINARY TRACT INFECTION?

Wipe genitals from front to back after using the toilet to avoid that bacteria enter the urinary tract.

Change pads and clean genitals if soiled.

Keep the genital area **clean** and **dry**.

Drink **plenty** of water.

Try to **move around** as much as possible

Grant Agreement number 900024

This project was funded by the European Union's Health Programm (2014-2020)

Co-funded by the Health Programme of the European Union

HAPPY PATIENT

Grant Agreement number 900024

3rd Health Programme

Co-funded by
the Health Programme
of the European Union

This project is funded by the
European Union's Health Programme (2014-2020)

The content of this leaflet represents the views of the author only and is his/her sole responsibility; it cannot be considered to reflect the views of the European Commission and/or the Health and Digital Executive Agency (HaDEA), replacing the former CHAFEA since 01 April 2021, or any other body of the European Union. The European Commission and the Agency do not accept any responsibility for use that may be made of the information it contains.

www.happypatient.eu

HAPPY PATIENT

5 myths about urinary tract infections (UTI) in nursing home residents

MYTH #1

A positive test (dipstick/culture) in the elderly always means presence of a UTI...

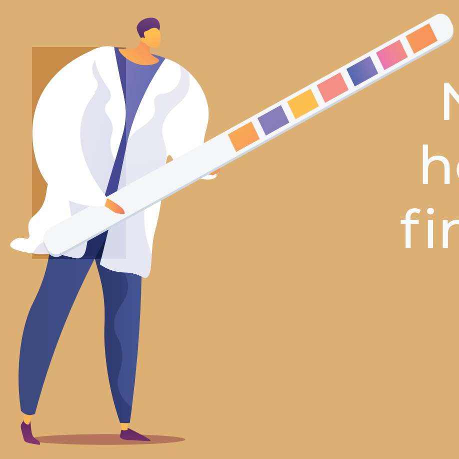

No, a substantial number of nursing home residents have bacteriuria and finding bacteria in the urine does not necessarily mean that the patient has a UTI.

MYTH #2

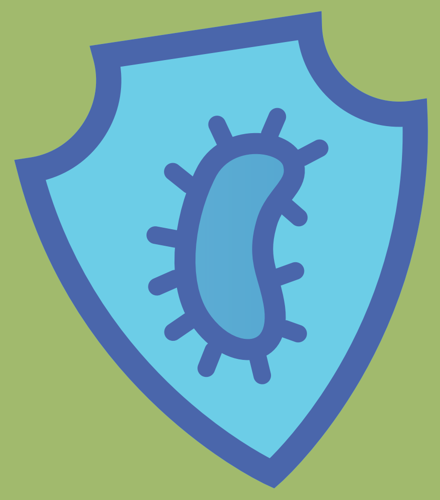

Antimicrobial resistance is not a problem in nursing homes...

There is a high prevalence of resistant bacteria in nursing homes and it may reduce the efficacy of antibiotic treatments.

MYTH #3

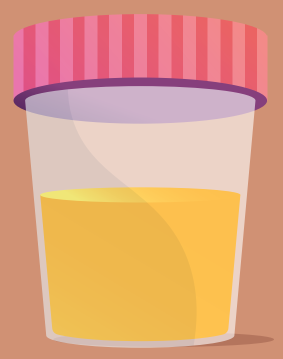

A single urinary symptom indicates high probability of UTI...

No. Only about half of patients with a single urinary symptom do have a UTI and to diagnose a UTI the urine should always be examined.

MYTH #4

Cognitive changes, agitation and confusion indicate high probability of UTI...

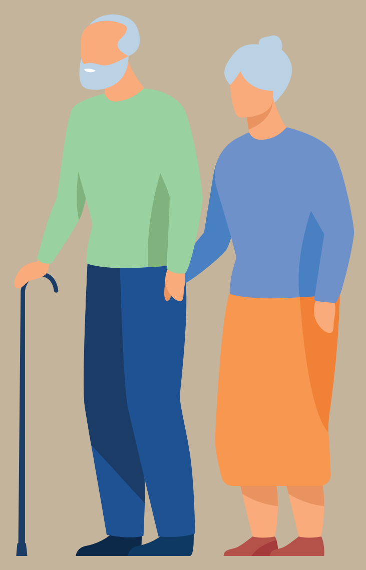

There are several possible causes of cognitive changes in the elderly, but UTI is not the most frequent one. Explore all possible causes, such as dehydration, pain, constipation, UTI, etc.

MYTH #5

When unsure of whether to prescribe an antibiotic or not ("better be safe than sorry"), the benefits of prescribing outweigh the harms of exposure to antibiotics...

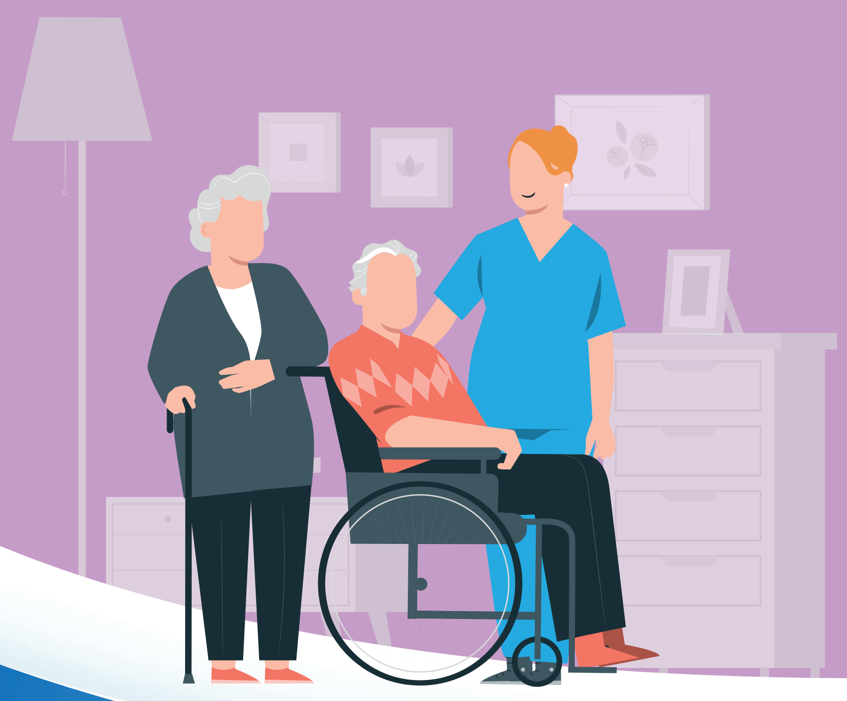

All antibiotics have side effects and may cause more harm than benefit particularly in the elderly people.

Additionally, all use of antibiotics can lead to antimicrobial resistance. This means that antibiotics become ineffective and infections become increasingly difficult to treat.

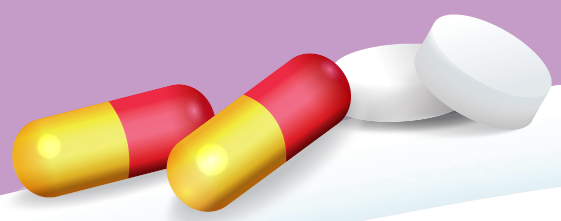

Co-funded by the Health Programme of the European Union

This project is funded by the European Union's Third Health Programme (2014-2020).
The content of this leaflet represents the views of the author only and is his/her sole responsibility; it cannot be considered to reflect the views of the European Commission and/or the Health and Digital Executive Agency (HaDEA), replacing the former CHAFEA since 01 April 2021, or any other body of the European Union. The European Commission and the Agency do not accept any responsibility for use that may be made of the information it contains.

HAPPY PATIENT

What you need to know if you have been prescribed an antibiotic

When to take your antibiotic:

- in the morning
- in the afternoon
- in the evening
- Shortly before sleeping
- Every ____ hours

How to take your antibiotic:

- At least 1 hour before or 2 hours after food
- After a meal or a snack
- Do not take with alcohol
- Do not take with dairy products
- In an upright or sitting down position
- Avoid sun exposure

Duration of antibiotic treatment:

You should stop your antibiotic treatment after ____ days.

Possible side effects include:

Diarrhoea, nausea and vomiting, abdominal pain, loss of appetite, skin rashes, headache, dizziness, fungal infections (candida).

Possible food and drug interactions:

- Combining the use of antibiotics with other medications or alcohol can modify the efficacy and increase the risk of adverse reactions.
- Ask your pharmacist or healthcare provider for any potential food and drug interactions.

**Most side effects are mild and temporary.
If side effects become severe, you should contact a healthcare professional**

Please remember:

- Antibiotics are only needed to treat certain infections caused by bacteria. Use of antibiotics can lead to antimicrobial resistance. This is why we need to use antibiotics only when necessary.
- Take the prescribed antibiotic treatment according to the prescription from the doctor.
- Don't share antibiotics with your family members, friends, or animals.
- Return any unused antibiotics to the pharmacy.
- Contact your healthcare provider if your symptoms get worse.
- Inform your healthcare provider and/or your pharmacist about any allergies you may have.
- If you are pregnant, breastfeeding, or planning to get pregnant, make sure that your healthcare provider and/or pharmacist is aware of it, to ensure safe use of the medication.

When to contact a pharmacist:

- For any questions about the medication you use.
- For information about relief of symptoms or side effects.
- If you are unsure about any of the information you have received.
- If you are unsure about how or when to take your medication.

HAPPY PATIENT

Co-funded by the Health Programme of the European Union

This project is funded by the EuropeanUnion's Third Health Programme (2014-2020).
The content of this leaflet represents the views of the author only and is his/her sole responsibility; it cannot be considered to reflect the views of the European Commission and/or the Health and Digital Executive Agency (HaDEA), replacing the former CHAFEA since 01 April 2021, or any other body of the European Union.The European Commission and the Agency do not accept any responsibility for use that may be made of the information it contains.

HOW DOES ANTIMICROBIAL RESISTANCE OCCUR?

There is usually a small number of bacteria that mutate and become resistant to antibiotics.

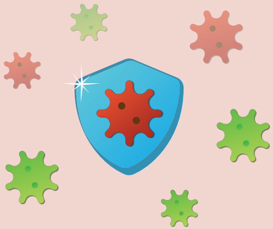

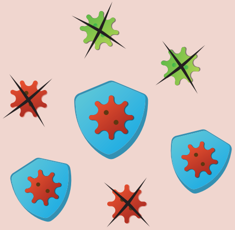

When we take antibiotics, they kill all susceptible bacteria, including the “good” bacteria that protect the body from infection. The drug-resistant bacteria survive and multiply.

Eventually, the drug-resistant bacteria become dominant and antibiotics stop working.

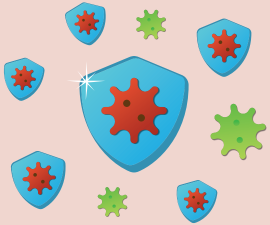

INAPPROPRIATE USE OF ANTIBIOTICS LEADS TO ANTIMICROBIAL RESISTANCE

This is a serious global challenge. That means that the antibiotics we have today will be less effective in the future in treating life-threatening infections.

Currently antimicrobial resistance is affecting millions of people worldwide and kills more than 1 million people per year.

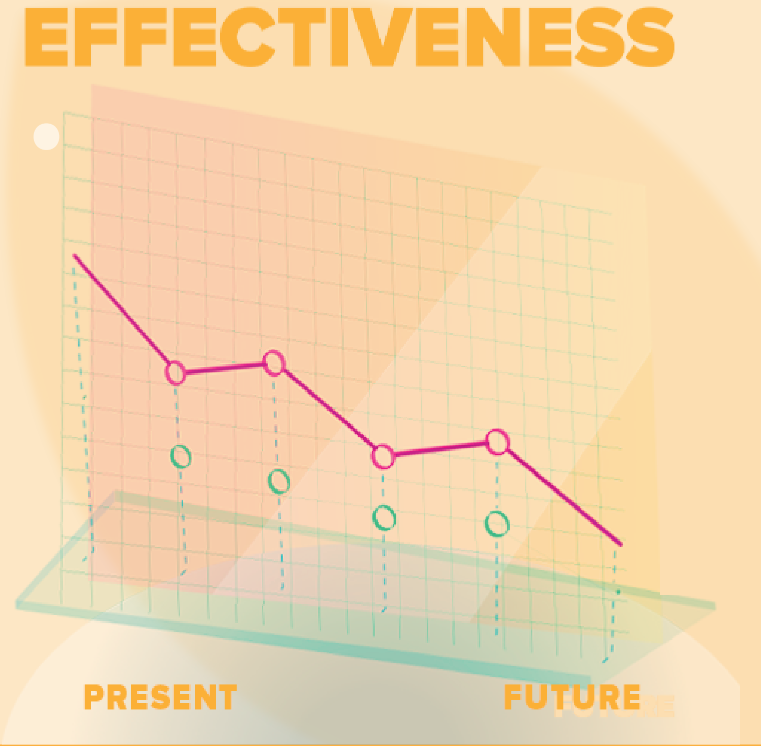

ANTIMICROBIAL RESISTANCE IS A GLOBAL PROBLEM

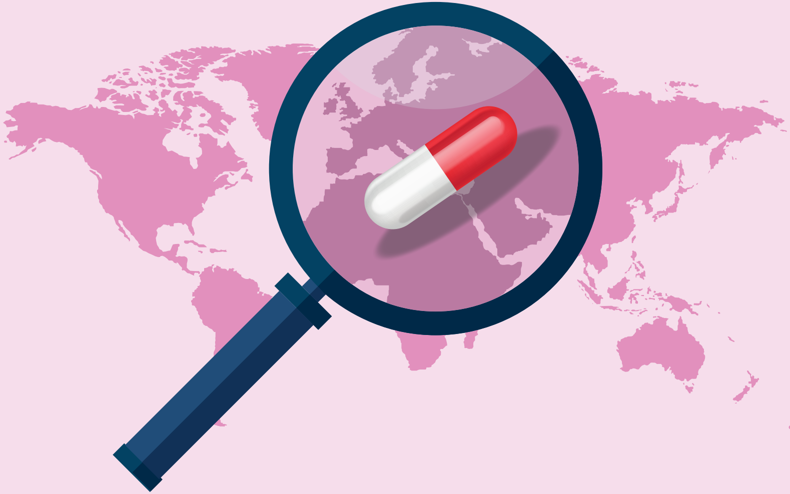

Everybody can contribute to the problem of antimicrobial resistance. Resistant bacteria do not respect borders.

SCAN ME

Do you want to know your own contribution to antimicrobial resistance?

WE NEED TO PROTECT AND PRESERVE OUR CURRENT ANTIBIOTICS

The problem of antimicrobial resistance will not be solved with new antibiotics.

Bacteria are smarter than humans and they become resistant to antibiotics shortly after using them.

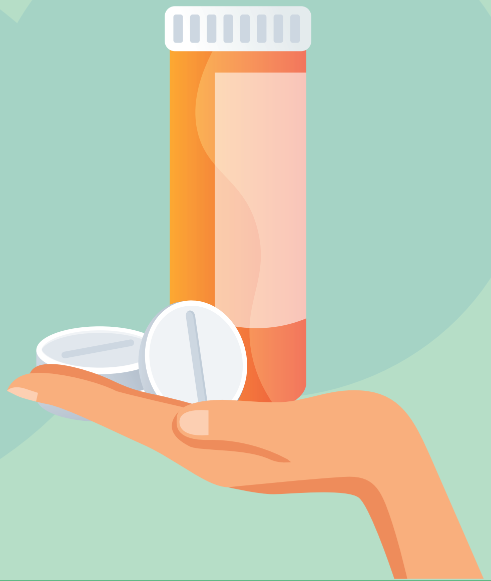

ANTIBIOTICS WON'T TREAT YOUR COLD OR FLU

Cold and flu are caused by viruses. Antibiotics are only effective against infections caused by bacteria, therefore they do not help against cold and flu.

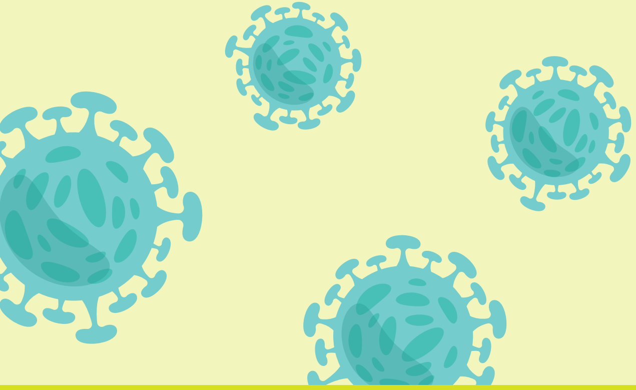

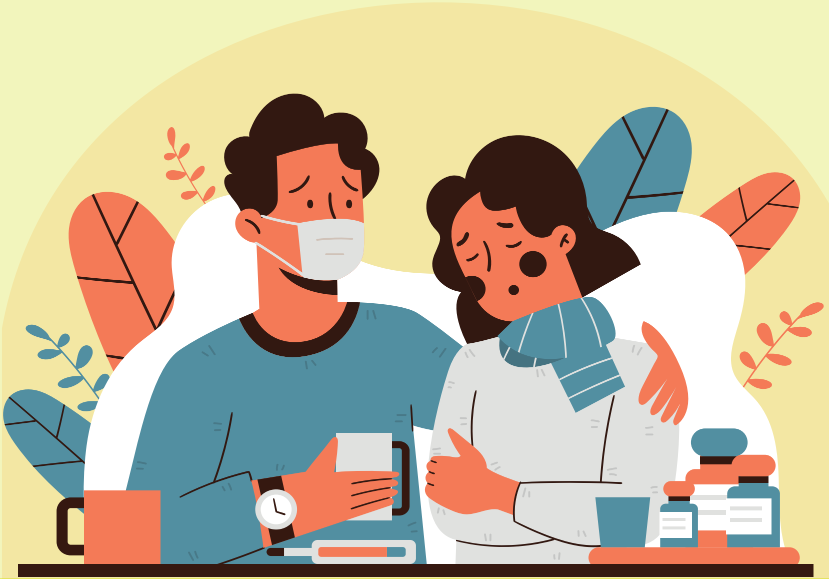

ANTIBIOTICS HAVE SIDE EFFECTS

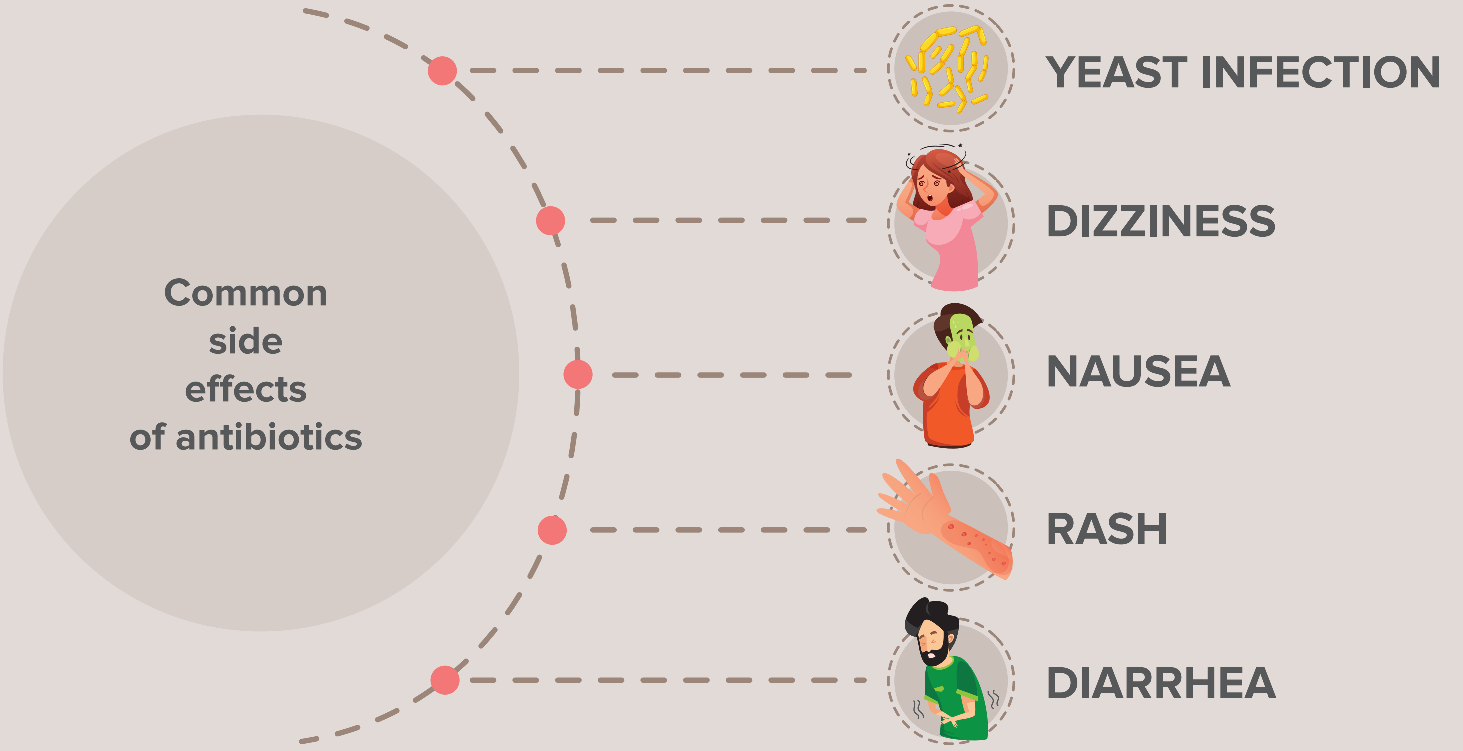

NEVER TAKE LEFTOVER ANTIBIOTICS OR SHARE ANTIBIOTICS WITH FAMILY & FRIENDS

The pharmacist can assess your symptoms, give you advice, recommend over-the-counter medication or tell you to contact a doctor.

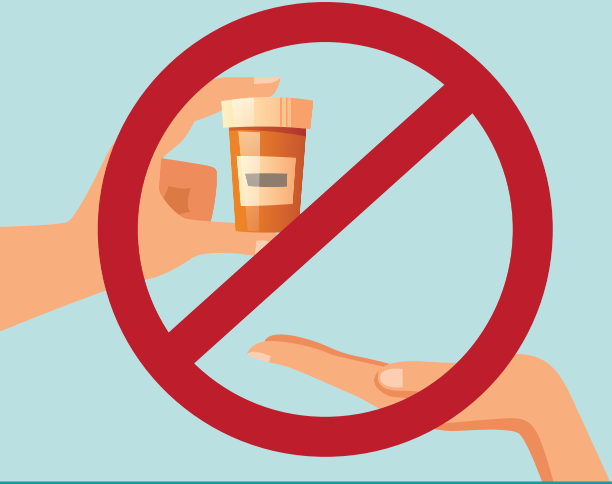

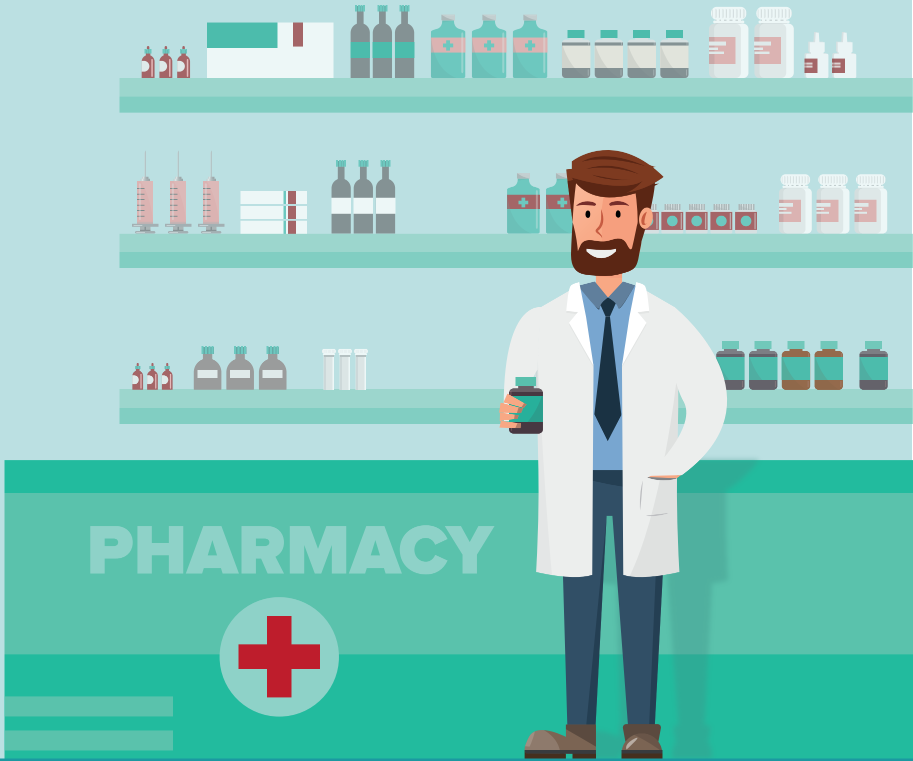

MORE INFORMATION ABOUT HAPPY PATIENT

HAPPY PATIENT

TRAINING HEALTHCARE PROFESSIONALS AND EMPOWERING THE PATIENT

SCAN ME and access our website and material

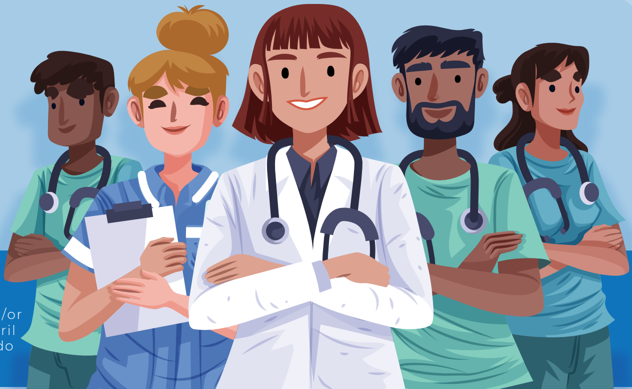

This project is funded by the European Union's Third Health Programme (2014-2020). The content of this poscard represents the views of the author only and is his/her sole responsibility; it cannot be considered to reflect the views of the European Commission and/or the Health and Digital Executive Agency (HaDEA), replacing the former CHAFEA since 01 April 2021, or any other body of the European Union. The European Commission and the Agency do not accept any responsibility for use that may be made of the information it contains.

Co-funded by the Health Programme of the European Union
