# Supplementary material for: Misconceptions and Knowledge Gaps on Antibiotic Use and Resistance in Four Healthcare Settings and Five European Countries—A Modified Delphi Study
Source: Antibiotics (Basel). 2023 Sep 11;12(9):1435. doi: 10.3390/antibiotics12091435 (PMC10525245; doi:10.3390/antibiotics12091435)
Supplement: Supplementary file 1 [file antibiotics-12-01435-s001.zip › Supplementary file S3.pdf]

Supplementary file S3 - List of project partners

No	Type	Role	Name	Short name	Country
1	Research org.	Clinical partners	Research Unit for General Practice Odense	RUPO	DK
2	Research org.		NORCE Norwegian Research Centre AS	NORCE	NO
3	Governmental		The Capital Region of Denmark	CAPREG	DK
4	Research org.		Rijksuniversiteit Groningen	RUG	NL
5	Affiliated to ICS	Host partners in the Target Countries	Fundacio Institut Universitari Pera la Recerca a l'atencio Primaria De Salut Jorgi Gol i Gurina	IDIAP	ES
6	University		Nice University Hospital	CHUNICE	FR
7	SME		My Family Doctor (Mano Seimos Gydytojas)	FDC	LT
8	University		Medical University of Lodz	MUL	PL
9	University		University of Crete	UOC	EL
