# Supplementary material for: Misconceptions and Knowledge Gaps on Antibiotic Use and Resistance in Four Healthcare Settings and Five European Countries—A Modified Delphi Study
Source: Antibiotics (Basel). 2023 Sep 11;12(9):1435. doi: 10.3390/antibiotics12091435 (PMC10525245; doi:10.3390/antibiotics12091435)
Supplement: Supplementary file 1 [file antibiotics-12-01435-s001.zip › Supplementary file S4 .pdf]

Supplementary file S4 – Delphi questionnaire with the list of the 44 statements

Dear participant,

Thank you very much for confirming your participation in the co-design of the intervention material for the HAPPY PATIENT project!

We greatly appreciate your willingness to serve **as an expert** in the process of **prioritizing importance to address the frequent knowledge gaps/misconceptions that affect the communication between you and the patient when discussing the need and use of antibiotics**. Your involvement in this process is crucial to ensure the feasibility and acceptability of those messages aiming to address these knowledge gaps/misconceptions in the context of your country and area of expertise.

This is the first prioritization Delphi round where you will be asked to rate a total of **44 WRONG statements** based on their relevance to address them in your opinion, taking into account the situation in your day-to-day practice. More specifically, the statements in this questionnaire represent some **frequent knowledge gaps/misconceptions.** These knowledge gaps/misconceptions were identified based on existing scientific literature and a context analysis conducted by our partners in the HAPPY PATIENT consortium. The prioritized statements will be addressed in the communication material (e.g. posters, leaflets) that will be produced for the HAPPY PATIENT project.

The questionnaire is divided into **4 themes**:Knowledge gaps/misconceptions specific to antimicrobial resistance (AMR) Knowledge gaps/misconceptions about the use of antibiotics for common community-acquired infections in general (CAIs)Knowledge gaps/misconceptions about the use of antibiotics for respiratory tract infections (RTIs)Knowledge gaps/misconceptions about the use of antibiotics for urinary tract infections (UTIs).

Please, note that some of the identified knowledge gaps/misconceptions should be interpreted by the point of view of the patients. Those are clearly specified in brackets, next to the statements. Time expected to be spent on the questionnaire is 20-30 minutes and should be filled in one occasion. Please, note that after submitting the questionnaire you will not be able to revise your answers. However, it is possible while filling in to go back to previous answers and change them before submitting the survey.

In the beginning of the questionnaire, you will be asked to provide the country and sector that you are representing, as well as to provide your email address **twice.** It is important to have your correct e-mail address because as part of the process you will receive **personalized feedback**, where you can see how your responses compare with the responses of the rest of the panel of experts. All responses are confidential and will be stored in drives owned by the University of Copenhagen (UCPH) and only the team of researchers involved in the study will have access to them. All data will be used solely for the purposes of the study. Consent to participating in the study is obtained by completing the questionnaire. You have the right to withdraw from the study at any given time.

What do you have to do?

1. Read the wrong statement pointing at patients' and/or healthcare professionals' knowledge gaps/misconceptions.
2. Provide your opinion **on the degree of relevance of the knowledge gap/misconception during the communication between you and the patient when discussing the need of antibiotics.**
3. Rate your opinion on the following 5-point Likert scale: 1- Not important, 2- Little important, 3- Neutral/I don't know, 4- Important, 5- Very important.

Thank you again for your time!
Best regards,
The HAPPY PATIENT team

Please, indicate your email address.

_____

Please, verify your email address.

_____

Which sector are you representing?

(1) General Practice (Primary care clinic)

(2) Out-of-hours services (Secondary care clinic, Private practice)

(3) Nursing homes

(4) Community pharmacy

Which country are you representing?

(1) France

(2) Greece

(3) Lithuania

(4) Poland

(5) Spain

Theme 1. Knowledge gaps related to antimicrobial resistance (AMR).

Based on your daily practice and experience, how important do you believe it is to address the below WRONG statements related to AMR?

	Not important	Little important	Neutral / I don't know	Important	Very important
1. I cannot contribute to the increase of antimicrobial resistance. [From the point of view of the patient]					
2. Others, not me, are responsible for controlling the problem of antimicrobial resistance.					
3. Antimicrobial resistance is not an important problem because better antibiotics are continuously being discovered.					
4. If I am not exposed to antibiotics, then I cannot carry or transmit antibiotic-resistant bacteria. [From the point of view of the patient]					
5. Bacteria resistant to antibiotics are only present in hospitals. [From the point of view of the patient]					
6. Antimicrobial resistance is not a problem in my country. [From the point of view of the patient]					

7. Antimicrobial Resistance is not a problem where I work (i.e., general practice, out-of-hour services, nursing homes, pharmacies).	
8. Not all antibiotics are at risk of becoming ineffective against infections by resistant bacteria. [From the point of view of the patient]	

Theme 2. Knowledge gaps related to the use of antibiotics in community-acquired infections (CAIs) in general.

Based on your daily practice and experience, how important do you believe it is to address the below WRONG statements related to the use of antibiotics when diagnosing and managing community-acquired infections (in general)?

	Not important	Little important	Neutral / I don't know	Important	Very important
9. Antibiotics are effective against all type of infections. [From the point of view of the patient]					
10. Broad spectrum antibiotics, such as quinolones and 3rd - 5th generation cephalosporines, are the best treatment options because they cover a wide range of bacteria.					
11. A good doctor is the one that prescribes the newest type of antibiotics.					
12. Ending the consultation without an antibiotic prescription indicates that the doctor is not taking my symptoms seriously enough. [From the point of view of the patient]					
13. Ending the consultation without an antibiotic prescription, when the patient is asking for it, indicates lack of empathy from the doctor. [From the point of view of the patient]					

14. The single presence of fever suggests high probability of bacterial infection and need of antibiotics. [From the point of view of the patient]					
15. Ciprofloxacin, doxycycline, levofloxacin, ofloxacin, tetracycline, trimethoprim do not cause sensitivity to sunlight.					
16. The benefits of prescribing antibiotics when unsure of the bacterial or viral origin of the symptoms outweigh the harms of exposure to antibiotics.					
17. It is fine to use leftover antibiotics without consulting a healthcare professional, when experiencing similar symptoms to previous acute infections. [From the point of view of the patient]					

Theme 3. Knowledge gaps related to the use of antibiotics in respiratory tract infections (RTIs).

Based on your daily practice and experience, how important do you believe it is to address the below WRONG statements related to the use of antibiotics in respiratory tract infections?

	Not important	Little important	Neutral / I don't know	Important	Very important
18. The single presence of tonsillar exudate in					

patients with sore throat suggests a high probability of bacterial infection and need of antibiotics.	
19. The single presence of tender anterior cervical adenopathy in patients with sore throat suggests a high probability of bacterial infection and need of antibiotics.	
20. The majority of patients with a sore throat require antibiotic treatment.	
21. In patients with sore throat and other symptoms such as tonsillar exudates, fever, tender anterior cervical adenopathy, antibiotics have a great impact in the course of symptoms by shortening the length of symptoms by more than two days.	
22. All children with middle ear inflammation and ear pain require antibiotic therapy.	
23. A patient with the combination of two or more of the following symptoms : a) nasal congestion, b) nasal discharge, c) pain in the face/teeth, d) reduced sense of smell, e) fever; requires antibiotic therapy independently of the number of days with symptoms.	

24. A bacterial infection is the most common cause of the single or combined presentation of the following symptoms: a) nasal congestion, b) nasal discharge, c) pain in the face/teeth, d) reduced sense of smell, e) fever.	
25. A sinus X-Ray can help doctors to discriminate the bacterial or viral origin of the rhinosinusitis symptoms.	
26. Purulent nasal discharge suggests a high probability of bacterial infection and need of antibiotics.	
27. Based on the characteristics of the cough the health care professional can differentiate the viral or bacterial origin of the cough. For example, a chesty cough (wet, productive or phlegmy) means that it is caused by a bacterium.	
28. More than 2 weeks coughing suggests a high probability of bacterial infection and need of antibiotics.	
29. The presence of cough without other symptom suggests a high probability of bacterial infection and need of antibiotics.	
30. Cough with purulent sputum suggests a high	

probability of bacterial infection and need of antibiotics.					
31. Macrolides are the best first option for treating a bacterial lower respiratory tract infection in order to cover typical and atypical pathogens.					
32. As soon as I feel symptoms like sore throat, running nose, fever I should seek medical care to get antibiotics. [From the point of view of the patient]					

Theme 4. Knowledge gaps related to the use of antibiotics in urinary tract infections (UTIs).

Based on your daily practice and experience, how important do you believe it is to address the below WRONG statements related to the use of antibiotics in urinary tract infections?

	Not important	Little important	Neutral / I don't know	Important	Very important
33. The single presence of painful discharge of urine suggests a high probability of bacterial infection and need of antibiotics.					
34. The single presence of frequent urination suggests a high probability of bacterial infection and need of antibiotics.					
35. The single presence of burning sensation during urination suggests a high probability of bacterial					

infection and need of antibiotics.	
36. The single presence of persistent urge to urinate suggests a high probability of bacterial infection and need of antibiotics.	
37. The single presence of smelly urine suggests a high probability of bacterial infection and need of antibiotics.	
38. The single presence of cloudy urine suggests a high probability of bacterial infection and need of antibiotics.	
39. The single presence of blood in urine suggests a high probability of bacterial infection and need of antibiotics.	
40. When a patient comes with acute UTI symptoms it is okay to prescribe antibiotics, despite of the negative result of a dipstick test [nitrites (-), leucocytes (-)]. A negative dipstick test is not a good predictor of absence of UTI.	
41. In an uncomplicated UTI, antibiotic treatment should be started as soon as possible to prevent the dissemination of the infection to the kidneys and bloodstream, independently of the risk of complication.	

42. Cognitive changes (e.g. agitation, confusion) in the elderly suggest a high probability of bacterial infection and the need of antibiotics, even without the presence of urinary tract symptoms.	
43. A positive dipstick in the elderly without urinary tract symptoms is a strong indicator for urinary tract infection and requires antibiotics.	
44. Leucocytes positive and nitrite negative result in a dipstick test indicates with high certainty bacterial infection and need of antibiotics.	

Thank you very much for taking the time to fill in this questionnaire!
